# Pilot Feasibility Study of a Hospital-Based Post-Natal Educational Intervention on New Mothers in a BFHI-Compliant Tertiary Referral Center for Neonatal Care

**DOI:** 10.3390/ijerph19042020

**Published:** 2022-02-11

**Authors:** Alessandra Consales, Lorenzo Colombo, Lidia Zanotta, Daniela Morniroli, Patrizio Sannino, Serena Rampini, Giulia Piccoli, Michaela Donghi, Paola Marchisio, Fabio Mosca, Laura Plevani, Maria Lorella Giannì

**Affiliations:** 1 Department of Clinical Sciences and Community Health, University of Milan, Via della Commenda 19, 20122 Milan, Italy; alessandra.consales@unimi.it (A.C.); giuls.piccoli@yahoo.com (G.P.); michaela.donghi@gmail.com (M.D.); fabio.mosca@unimi.it (F.M.); maria.gianni@unimi.it (M.L.G.); 2 Fondazione IRCCS Cà Granda Ospedale Maggiore Policlinico, NICU, Via della Commenda 12, 20122 Milan, Italy; lorenzo.colombo@policlinico.mi.it (L.C.); lidia.zanotta@policlinico.mi.it (L.Z.); laura.plevani@policlinico.mi.it (L.P.); 3 Direzione Professioni Sanitarie, Fondazione IRCCS Cà Granda Ospedale Maggiore Policlinico, Via Francesco Sforza 28, 20122 Milan, Italy; patrizio.sannino@policlinico.mi.it (P.S.); serena.rampini@unimi.it (S.R.); 4 Fondazione IRCCS Cà Granda Ospedale Maggiore Policlinico, Pediatric Unit, 20122 Milan, Italy; paola.marchisio@unimi.it; 5 Department of Pathophysiology and Transplantation, University of Milan, 20122 Milan, Italy

**Keywords:** Baby-Friendly Hospital Initiative, breastfeeding, maternal education, post-partum hospital stay

## Abstract

The immediate post-partum period offers a valuable opportunity for parental education on various health topics. The aim of this study was to pilot test the feasibility in a tertiary referral center for neonatal care of a post-natal educational intervention (the Diary) designed to provide mothers with basic information concerning newborn care and breastfeeding. Furthermore, we aimed to evaluate its effect on exclusive breastfeeding rates at discharge and at 48 h post-discharge, and on maternal perceived support during hospital stay, compared to standard care. A single-center two-phase interventional study was carried out from 1 December 2018 to 2 June 2019. The Diary was given to mothers enrolled in Phase 2, together with the Nurse–Parent Support Tool (NPST). The Diary–NPST couples analyzed were 269. The Diaries filled out and returned were 62.2%. Overall, mothers rated the information received through the Diary as “clear and comprehensive”. Exclusive breastfeeding rates at discharge resulted in being higher in Phase 1 than in Phase 2 (80.6% vs. 72.5%, *p* = 0.04), whereas no difference emerged in terms of exclusive breastfeeding rates at 48 h. In both phases, the median NPST total score (4.05) was high. In conclusion, we propose a new instrument of in-hospital post-natal maternal education and, in line with the current literature, we support well-designed written educational materials to promote mothers’ knowledge and satisfaction with post-partum hospital assistance. Further studies that are multicentric and with a longer follow-up period are needed to evaluate the potential impact of the Diary on exclusive breastfeeding duration.

## 1. Background

Among the minimum criteria for the discharge of the healthy-term newborn, the American Academy of Pediatrics (AAP) Committee on Fetus and Newborn includes that the mother has received training and demonstrated competency in the care of her newborn [1]. Consequently, strengthening mothers’ knowledge and confidence and fostering the development of parenting skills are two key aspects of discharge planning of a newborn from the post-natal unit.

Human milk is recognized as the optimal feeding for virtually all infants because of its renowned health benefits to the dyad [2]. However, although commonly considered natural and physiological, successful breastfeeding is still an acquired skill that needs to be learned [3]. Breastfeeding knowledge and self-efficacy are considered modifiable factors closely related to breastfeeding prevalence and duration [4]. Several studies have been conducted over the years to investigate breastfeeding educational programs, both ante-natal [5] and post-natal [4], in a hospital setting [6], at home [7] or smartphone-based [8], through group or individual sessions [5,9]. However, the most effective and time-appropriate way to deliver breastfeeding educational interventions is still being debated [10]. We therefore decided to implement in our Baby Friendly Hospital Initiative (BFHI)-compliant hospital a post-natal educational intervention (hereinafter referred to as the Diary) designed to provide mothers with basic written information concerning newborn care and breastfeeding, in addition to the standard care and support regularly offered to every dyad.

The primary aim of the present study was to pilot test the feasibility of the Diary in a tertiary referral center for neonatal care. We furthermore aimed to evaluate its effect on exclusive breastfeeding rates at discharge and at 48 h post-discharge, and on maternal perceived support from healthcare professionals during hospital stay, compared to standard care.

## 2. Materials and Methods

### 2.1. Study Design and Setting

A single-center two-phase interventional study was carried out in the post-natal unit of our hospital, a BFHI-compliant facility. Our hospital, located in Milan (Lombardy, Italy), is a tertiary referral center for neonatal care with around 6000 births per year. Our neonatology unit is the largest in Italy and among the largest in Europe. Consequently, the hospital has a wide catchment area that, while including mainly mothers from Lombardy, also extends beyond regional borders.

In our post-natal unit, we promote and support breastfeeding in all mother–infant dyads throughout hospital stay. All healthcare professionals, from neonatologists to nurses to an International Board-Certified Lactation Consultant (IBCLC), are actively involved in the promotion of breastfeeding and support at the bedside. Our policy is described in a written breastfeeding protocol, which is based on the BFHI [11] and the Ten Steps to Successful Breastfeeding [12].

Phase 1 of the present study was conducted from 1 December 2018 to 1 March 2019; Phase 2 was conducted from 2 March 2019 to 2 June 2019.

During Phase 1, a control group of usual care patients received routine post-partum hospital education (i.e., breastfeeding and peri-partum care, on demand one-on-one breastfeeding assistance, educational videos on infant care and pre-discharge group education session).

During Phase 2, a maternal educational intervention was implemented in addition to routine care.

During both phases, the Nurse–Parent Support Tool (NPST) was administered to enrolled mothers.

The present study followed the Declaration of Helsinki code of conduct and was approved by the Ethics Committee of Fondazione IRCCS Ca’ Granda Ospedale Maggiore Policlinico (22 November 2018). Written informed consent was obtained from all mothers enrolled and from both parents for use of neonatal data.

### 2.2. Study Sample

During both phases, participation in the present study was proposed to all mothers who gave birth to healthy-term newborns at our hospital and had a good oral and written comprehension of the Italian language. Exclusion criteria for both phases were inadequate oral and/or written comprehension of the Italian language, mothers of newborns hospitalized in the Neonatal Intensive Care Unit (NICU), mothers with contraindications to breastfeeding (i.e., previous breast surgery, drugs incompatible with breastfeeding, and HIV or human T-cell lymphotropic virus infection) and/or who had chosen not to breastfeed.

Mothers who did not return the Diary and/or NPST or returned an incomplete Diary and/or NPST were considered dropouts.

In the case of twins, only the first-born child was considered for the analysis of breastfeeding outcomes.

The sample size was based on the number of Diaries and NPSTs filled out during the selected time periods. Thus, the sample size was based on feasibility and availability, rather than power analysis.

A 48-h follow-up was performed by means of routine post-discharge visit. No patient was lost to follow-up.

### 2.3. Instruments

#### 2.3.1. Post-Natal Educational Intervention

The post-natal educational intervention was structured in the form of a self-filling Diary that was handed out during Phase 2 to all new mothers by a nurse either in the delivery room or upon their arrival in the post-natal unit. The Diary was presented to mothers as a newly implemented instrument aimed at boosting their knowledge on post-natal care and their confidence in taking care of their newborn. The nurse also explained to mothers that the Diary did not in any way substitute the healthcare personnel, who was always available to clarify any doubt they might have. The exact definitions [12] of skin-to-skin contact and rooming-in practice were also specified to mothers during this first interaction, in order to facilitate comprehension of the related sections in the Diary. In order not to compromise the authenticity of the responses, mothers were asked to fill out the Diary independently, without sharing their answers with their partners or fellow mothers. At discharge, a different member of the nursing staff collected the Diary.

The Diary was created by a multidisciplinary team composed of nurses, neonatologists and an IBCLC, following an extensive review of the existing literature on breastfeeding and newborn care. In particular, documents by WHO/UNICEF [13,14,15,16,17], Ministero della Salute (Italian Ministry of Health) [18] and National Health Service (NHS) [19,20], on which our institutional infant feeding policies are based, were taken into consideration.

The Diary addresses different topics and combines educational segments with questions on mothers’ personal experiences during the first days of the newborn’s life. The topics addressed are skin-to-skin contact, rooming-in, newborn’s feeding cues, breastfeeding, bowel movements and diuresis, and characteristics of breasts and nipples.

Short explanations have been included in the Diary regarding skin-to-skin contact, rooming-in, feeding cues, breastfeeding, bowel movements and diuresis. To facilitate understanding, explanatory tables and images have also been included in the text. In particular, the Diary features a visual representation of a newborn’s stomach and its variations over the first weeks of life [18], pictures of a newborn showing the most common hunger cues [18] and a table containing the normal parameters of bowel movements and diuresis (expressed as number and approximate weight of diapers worn) according to days of life [20].

At the end of each explanatory section, mothers are asked to judge the quality of the information received on the matter by stating one of the following: “it has been clear and comprehensive” or “I need more information”.

For the Diary to be an interactive instrument, thought-provoking multiple-choice questions have been added in the sections regarding skin-to-skin contact and rooming-in. Here, mothers are asked to specify time to first skin-to-skin contact and its duration, and to explain the reasons for a non-continuous rooming-in (if that has been the case). Aim of such questions is for mothers to compare the theory (i.e., what has been explained at the beginning of the sections) with their real-life experience. For the same reason, in the section about bowel movements and diuresis, a table has been added to help parents keep track of their newborn’s feedings, bowel movements and diuresis during the first three days post-partum.

In the final section of the Diary, a breastfeeding observation form (adapted from Reference [17]) has been added for mothers to fill out independently, to help them acquire greater awareness about the breastfeeding process. The form contains multiple-choice questions regarding general conditions of mother and newborn, position, latch-on and suckling of the newborn at the breast. A visual representation of the two breasts with a list of possible signs to check (e.g., breast fullness, nipple pain and redness) is also included.

In the last page of the Diary, a sentence written in bold reminds mothers to always reach out for the healthcare personnel if in doubt.

#### 2.3.2. The Nurse–Parent Support Tool

We used the NPST [21] to evaluate maternal perception of post-partum assistance. The NPST was given to mothers enrolled in Phase 1 and 2 by a nurse during hospitalization (day 2) and collected by a different nurse at discharge. At our hospital, mean length of stay for mothers of healthy newborns varies from 2 or 3 to 5 days, according to mode of delivery (vaginal vs. cesarean section, respectively).

In order not to compromise the authenticity of the responses, mothers were asked to fill out the NPST independently, without sharing their answers with their partners or fellow mothers.

The NPST is a 21-item questionnaire which investigates, on a 5-point Likert scale, the four domains of support, as identified by the Nurse–Parent Support Model (Table 1).

A mean total score is obtained by adding up the answers to the various questions. A higher total score corresponds to a greater perception of support from health professionals.

The questionnaire also includes two final open-ended questions encouraging mothers to specify any other way that health professionals have been of help, and to provide suggestions on how the dyad could be better assisted.

The Italian translation of the original NPST was edited by R. Montirosso and B. Premoli (IRCCS Medea, Association “Our Family”, Bosisio Parini LC, Italy, 2005) and validated on a sample of 25 Italian NICUs [22]. Montirosso and colleagues compared the original and the translated version of the NPST and revised the latter to reduce conceptual transcultural differences in item content. The authors concluded that the NPST had appropriate psychometric properties when tested on a wide sample of Italian mothers.

### 2.4. Data Collection

Obstetric medical records and infants’ computerized medical charts (Neocare i&t Informatica e Tecnologia Srl, Lecce, Italy) were used to obtain basic characteristics of mothers (i.e., age, ethnicity, level of education, mode of delivery and marital status) and newborns (i.e., gender, gestational age, birthweight and mode of feeding at discharge). All other data were obtained from the Diary and the NPST.

On the routine 48-h post-discharge visit, the mode of feeding was recorded.

The mode of feeding was classified according to the WHO definitions [2].

### 2.5. Statistical Analysis

Categorical variables were expressed as numbers (frequencies) and compared by using the χ^2^ test. Continuous variables were expressed as medians and tested between subgroups with the independent samples t-test and nonparametric tests, as appropriate.

A *p* < 0.05 was considered statistically significant.

Statistical analysis was performed with SPSS version 25 statistic software package (SPSS Inc., Chicago, IL, USA).

## 3. Results

**Figure 1:**
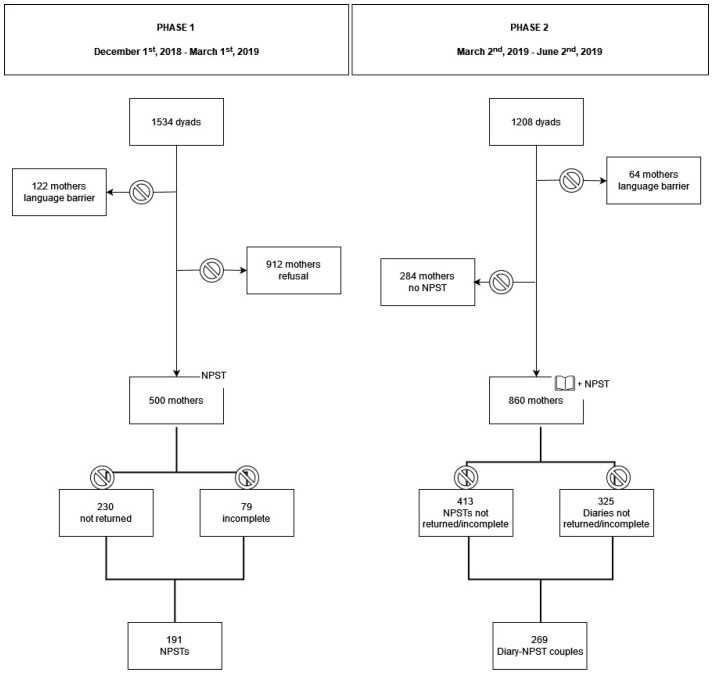
Participants’ flow through study.

Figure 1 outlines participants’ flow through the study.

The total eligible population for Phase 1 included 1534 mother–infant dyads admitted to the post-natal unit of our hospital from 1 December 2018 to 1 March 2019. A total of 122 dyads were excluded for insufficient understanding of the Italian language, and 912 mothers refused to participate in the study. Of the 500 NPSTs administered, 230 were not returned and 79 were eliminated because they were incompletely filled out. The Phase 1 NPSTs analyzed for the purposes of the present study amounted to 191 (attrition rate 61.8%). However, since 270 NPSTs were returned (although 79 of them were incomplete), the adherence rate was 54%.

The total eligible population for Phase 2 included 1208 mother–infant dyads admitted to the post-natal unit of our center from 2 March 2019 to 2 June 2019. Sixty-four dyads were excluded for insufficient understanding of the Italian language and 284 because they refused to fill the NPST. Of the 860 Diary–NPSTs administered, 413 NPSTs and 325 Diaries were not returned or were eliminated because they were incompletely filled out. The 447 NPSTs collected were subsequently paired with the 535 Diaries returned: 178 NPSTs did not have the corresponding Diary. The Diary–NPST couples that were analyzed for the purposes of the present study amounted to 269 (attrition rate 68.7%). However, since the Diaries that were completely filled out and returned were 535/860 (62.2%), adherence to the Diary was moderate–high.

The basic characteristics of the study population are summarized in Table 2. The comparison between the two phases shows that mothers enrolled in Phase 1 were of foreign origin (i.e., not Italian) in a greater percentage of cases than mothers enrolled in Phase 2 (14.7% vs. 8.9%, *p* = 0.05). Moreover, mothers enrolled in Phase 2 were older (35 (32–38) vs. 34 (30–37) years, *p* = 0.01), and had undergone cesarean section more often than mothers in Phase 1 (37.2% vs. 31.9%, *p* = 0.0005). The remaining maternal sociodemographic and clinical characteristics were superimposable between the two phases. The clinical characteristics of the neonatal population (i.e., sex, gestational age and birthweight) did not differ significantly between the two phases.

### 3.1. The Diary

Data obtained from the Diaries are summarized in Table 3.

In most cases (51.72%), the Diary was handed out to mothers directly in the delivery room. The answers to the multiple-choice questions in the first section of the Diary showed that almost all dyads (93.59%) practiced skin-to-skin contact after birth. In 64.85% of cases, skin-to-skin contact began within 5 min of delivery, and it lasted at least 60 min in 76.12% of cases. Regarding rooming-in, 64.75% of mothers stated that they practiced continuous rooming-in (i.e., 23/24 h per day). The reasons why newborns were left in the nursery were grouped into three categories: “newborn’s clinical reasons” (43.08%), “maternal rest” (49.74%) and “both” (7.18%).

When asked to evaluate the quality of the information received through the Diary about skin-to-skin contact, the baby’s hunger cues, rooming-in and bowel movements and diuresis, overall, mothers described it as “clear and comprehensive” (94.01%, 94.93%, 91.74% and 92.9%, respectively).

The last part of the Diary was dedicated to the maternal self-evaluation of breastfeeding: 89.78% of mothers stated that they felt “relaxed and at ease” during breastfeeding, and only 9.49% reported feeling “tense and uncomfortable”. Regarding the newborn’s conditions, 90.53% of mothers wrote that their baby was in a state of general well-being during breastfeeding. Based on the responses to the breastfeeding observation form, it emerged that most mothers rated position (79.42%) and latch-on (75.45%) as adequate. Greater uncertainty (37.61%) emerged regarding the suckling of the newborn, but in this case, as well, more than half of the mothers (58.55%) reported it as being adequate.

As for breast observation, 34.44% of mothers did not notice any change during hospitalization; almost half of the women enrolled (49.79%) described a physiologic change, whereas 15.77% described an abnormal change. More than half of the study population (60.17%) reported experiencing nipple pain. Finally, 60.58% of women did not notice redness at the nipple, while 25.73% reported a “faded” redness and only 13.69% a “marked” redness.

### 3.2. Breastfeeding Outcomes

Exclusive breastfeeding rates at discharge and at 48 h post-discharge during the two phases are illustrated in Table 4.

Exclusive breastfeeding rates at discharge resulted in being higher in Phase 1 than in Phase 2, whereas no difference emerged between the two phases in terms of exclusive breastfeeding rates at 48 h. A 2.6% decrease in exclusive breastfeeding rates at 48 h was observed in Phase 1, compared to a 1.1% increase observed in Phase 2 in the same time-span.

## 4. The NPST

NPST Cronbach’s alfa for Phase 1 and Phase 2 was 0.929 and 0.941, respectively.

Table 5 compares the median NPST total scores of the whole population, Phase 1 and Phase 2. Table 5 also shows the median values reported for each support domain.

In both phases, the median NPST total score (4.05) was high, indicating a high perception of support. Items in the caregiving-support domain scored the highest median values, whereas the lowest median values were reported in items belonging to the emotional-support domain.

## 5. Discussion

Although attrition rate was high in both phases, the results of the present pilot feasibility study show that the modality chosen to deliver the educational intervention was generally well accepted by mothers, as demonstrated by the percentage of Diaries filled out and returned (535/860, 62.2%). Furthermore, most mothers described the information received through the Diary on various topics regarding breastfeeding and newborn care as clear and comprehensive. Exclusive breastfeeding rates at discharge and at 48 h post-discharge were not positively affected by the educational intervention, consistently with the notion that breastfeeding outcomes are bound, for their very nature, to be influenced by many more factors [23]. However, a 2.6% decrease in exclusive breastfeeding rates at 48 h was observed in Phase 1, compared to the slight increase observed in Phase 2. In both phases mothers’ overall perception of the quality of the support received by healthcare professionals during hospital stay was high, as indicated by the NPST scores reported.

The WHO/UNICEF Ten Steps to Successful Breastfeeding, which the BFHI relies on, stress the importance of in-hospital maternal support and education on the topic of breastfeeding [24]. In particular, Step 2 focuses on the importance of staff knowledge, competence and skills to provide adequate breastfeeding support; Step 5 underlines the need for healthcare professionals to help mothers manage common difficulties arisen during breastfeeding; and Step 8 prompts healthcare professionals to teach parents how to recognize and respond to their infants’ cues for feeding.

Breastfeeding education and support interventions aimed at improving maternal self-efficacy are heterogeneous in nature, and the optimal education modality and timing are still debated. In 2011, Hilmiye Aksu et al. [7] showed how breastfeeding education offered during the early post-partum period was effective in increasing breastfeeding knowledge and duration. Aksu’s educational intervention consisted in 30-min home visits by breastfeeding supporters on day 3 post-partum. In Turkey, the country where this randomized controlled trial was performed, all pregnant and lactating women are reportedly visited routinely by the midwives in their area. This option would be less feasible in other countries, such as Italy, where such home support is not routinely offered. The in-hospital immediate postpartum period was considered by Gao et al. [25] a significant time for healthcare workers to give professional support to mothers for establishing exclusive breastfeeding. Indeed, the post-partum hospital stay offers a valuable “window of opportunity” for parental education [26,27], since, during this short period of time, mothers are generally eager to learn as much as they can about newborn care. However, brief postpartum hospital stays (at our hospital, the mean hospital stay for mothers of healthy newborns varies from 2 or 3 to 5 days, according to mode of delivery, vaginal vs. cesarean section, respectively) often leave insufficient time for healthcare professionals to address a new mother’s learning needs effectively. Moreover, the high influx of patients at our hospital, which covers around 6000 pregnancies per year, may sometimes result in a skewed healthcare professional:dyad ratio that may limit the time available to interact with mothers and respond effectively to their learning needs. Our decision to create a written educational instrument was indeed partly based on the recognized need to provide every mother with the basic information concerning breastfeeding and newborn care, thus bypassing the relative shortage of time and medical and nursing staff. Evidently, the Diary is not a substitution for health personnel, who must remain available for the necessary clarifications and integrations. Moreover, assistance provided to the mother must be tailored, considering the many peculiarities of every dyad. However, given its written, and therefore durable, nature, the Diary has the advantage of giving mothers something to reference once they go home, usually far from healthcare professionals.

Since it has long been shown how mothers value written information [28,29], unsurprisingly several studies over time have adopted booklets to implement maternal educational interventions [6,30,31,32,33,34]. In particular, Kronborg et al. [31] created a booklet to provide useful information to post-partum mothers as part of their supportive intervention, aiming at assessing its impact on the duration of breastfeeding. Their booklet covered two topics: how to breastfeed and how to read the baby’s cues. However, the cornerstone of their intervention was home visits during the first 5 weeks post-partum, and not the booklet itself. More akin to our study, Buchko et al. [6] designed a quality improvement project based on a comprehensive education booklet, with the aim to improve the quality and efficiency of post-partum education during hospitalization. However, Buchko’s booklet was 60 pages long, and its comprehensiveness resulted in most new mothers reporting to have received more information than they needed. Our Diary was purposefully kept short and to the point. Moreover, it was not intended to be a booklet as much as an interactive instrument which mothers were asked to fill in (e.g., with their newborn’s bowel movements and diuresis or a description of the breasts or the newborn’s latch-on). We believe that this strategy prompts a greater maternal involvement, thus allowing mothers to become more than passive recipients of the information provided but, rather, be actively involved in the learning process. Furthermore, our Diary was intended to provide practical information and advice to teach mothers how to manage their newborn, what to monitor and how to tackle the most common challenges encountered during the first days of life of their newborn, thus facilitating a smooth transition into motherhood. Indeed, in order for mothers to feel confident in their parenting role, they must acquire knowledge and develop new skills [35]. Parental well-being and their sense of competence and self-efficacy in caring for the infant have been shown to influence the development of a healthy parent–child relationship and positive parenting [36], which are, in turn, related to the child’s socio-emotional, cognitive and behavioral development [37].

In the present study, the high NPST scores reported testified great maternal satisfaction with the assistance received at our center. The highest scoring items were those belonging to the caregiving and informational support categories. The lack of difference between the two phases suggests consistency in the assistance offered by our healthcare personnel, who strictly follows the BFHI indications, and which is therefore independent of the use of the Diary.

The main limitation of the present study is the lack of randomization, which likely resulted in two slightly skewed populations, with mothers in Phase 2 being older, more frequently Italian and recovering from a cesarean section than mothers in Phase 1. This may have at least partly contributed to the higher exclusive breastfeeding rates at discharge reported in Phase 1 compared to Phase 2 [38,39,40].

Likewise, since a sample size was not calculated a priori, the precision of our estimates and the power of our study to draw conclusions could have been affected. Thus, a sampling error cannot be excluded, and it can be speculated that the relatively small number of participants may have hidden potential differences between the two phases in terms of breastfeeding outcomes and/or NPST scores.

Moreover, attrition rate in both phases was high, although adherence to the instrument proposed (the Diary) was moderate–high. Consequently, an attrition bias cannot be excluded, and it may have influenced the results of the complete case analysis performed. In the present study, the reasons behind the dropouts were not investigated, but they shall be in future studies on the subject in order to better determine their impact on the results. Secondly, it would have been interesting to document possible differences in terms of breastfeeding rates between primiparas and multiparas. Likewise, the present study did not analyze mothers who underwent operative vaginal deliveries and/or delivered prematurely. Those dyads usually need extra support and encounter more difficulties in breastfeeding [41,42]. Said populations, hereby not considered due to the preliminary nature of our study, shall be included in future studies analyzing the impact of the Diary on perceived maternal support and exclusive breastfeeding rates.

Finally, since the present study describes the experience of a single Italian tertiary referral center for neonatal care, it may be flawed by a potential selection bias that might limit the reproducibility of our results in other settings. Further studies, maybe multicentric and with a longer follow-up period, would therefore be needed to evaluate the potential impact of the Diary on breastfeeding outcomes and perceived maternal support. We cannot exclude that a longer follow-up period could have highlighted a potential effect on exclusive breastfeeding duration. Indeed, several studies have shown that breastfeeding educational interventions impact breastfeeding duration [4,31,32] and that women with high self-efficacy are more likely to continue breastfeeding exclusively even when difficulties arise [43,44].

## 6. Conclusions

In conclusion, our study has the merit to have proposed a new instrument of in-hospital post-natal maternal education that has ever since become standard of practice at our hospital as part of the perinatal assistance offered at our post-natal unit, providing all mothers with the must-know information fundamental to transition smoothly into parenthood. Owing to its simplicity, yet comprehensiveness, we believe that our intervention can be easily replicated at other institutions seeking to promote maternal post-partum education on breastfeeding and basic principles of newborn care. Indeed, in line with the current literature [6], we support well-designed written educational materials as an effective and rather inexpensive way to promote new mothers’ knowledge and satisfaction with post-partum hospital assistance. Further studies that are multicentric and with a longer follow-up period are needed to evaluate the potential impact of the Diary on exclusive breastfeeding duration.

### Take-Home Messages

- The Diary was generally well accepted by mothers, who described the information received through it on breastfeeding and newborn care as clear and comprehensive. Such results prompted us to include the Diary as part of the perinatal assistance routinely offered at our post-natal unit;
- No difference in maternal perception of support from healthcare professionals during hospital stay emerged between the two phases. We consider this a positive result, since it suggests consistency in the assistance offered at our post-natal unit by our healthcare personnel, who strictly follows the BFHI indications, and which is therefore independent of the use of the Diary. The Diary should indeed be intended as an additional element of post-natal assistance, and not its core;
- Exclusive breastfeeding rates at discharge resulted in being higher in the mothers who did not receive the educational intervention (Phase 1). However, mothers in Phase 2 were older, more frequently Italian and recovering from a cesarean section than mothers in Phase 1. This, given the known lower exclusive breastfeeding rates reported in these populations, may be considered at least partly responsible for our results. More importantly, said difference in exclusive breastfeeding rates between the two phases lost statistical significance at 48 h. Therefore, a potential effect of the educational intervention in the long term cannot be excluded, also keeping in mind that the Diary consists of written material that mothers can reference once they get back home, usually far from healthcare professionals. Further studies, with a greater sample size and longer follow-up, are needed to confirm our hypothesis on a possible impact of the Diary on the duration of exclusive breastfeeding.

## Figures and Tables

**Table 1:** Four domains of support assessed by the NPST.

Domain of Support	Items
Informational support	NPST 2, NPST 3, NPST 6, NPST 7, NPST 8, NPST 9, NPST 10, NPST 14, NPST 16
Emotional support	NPST 1, NPST 12, NPST 13
Parental esteem support	NPST 4, NPST 5, NPST 11, NPST 18
Caregiving support	NPST 15, NPST 17, NPST 19, NPST 20, NPST 21

**Table 2:** Basic characteristics of study participants.

Variable	Total Population (*n* = 460)	Phase 1 (*n* = 191)	Phase 2 (*n* = 269)	*p*
**Sociodemographic characteristics**				
Age (years)	34 (31–37)	34 (30–37)	35 (32–38)	**0.01**
Foreign origin (%)	52 (11.3)	28 (14.7)	24 (8.9)	**0.05**
University degree/PhD (%)	309 (67.2)	124 (64.9)	185 (68.8)	0.38
Single parent (%)	18 (3.9)	7 (3.7)	11 (4.1)	0.81
**Clinical characteristics**				
Primiparity (%)	249 (54.1)	106 (55.5)	143 (53.2)	0.62
Cesarean section (%)	161 (35)	61 (31.9)	100 (37.2)	**0.0005**
**Neonatal characteristics**				
Male sex (%)	244 (53)	106 (55.5)	138 (51.3)	0.37
Gestational age (weeks)	39 (38–40)	39 (38–40)	39 (38–40)	0.56
Birth weight (g)	3305 (3060–3570)	3350 (3050–3590)	3300 (3080–3550)	0.62

*p* values < 0.05 are highlighted in bold.

**Table 3:** Data obtained from the 269 Diaries retrieved.

	**%**
**Diary Delivery**	
Delivery room	51.72
Post-natal unit	48.28
**Skin-to-Skin Contact**	
Yes	93.53
No	6.47
**Beginning**	
Within 5 min of birth	64.85
Between 3 and 60 min from birth	22.53
After 60 min from birth	12.63
**Duration**	
≥60 min	76.12
<60 min	23.88
**Quality of the information received**	
Clear and comprehensive	94.01
Need more information	4.93
**Newborn’s Feeding Cues**	
**Quality of the information received**	
Clear and comprehensive	94.93
Need more information	4.35
**Continuous Rooming-In**	
Yes	64.75
No	35.25
**Reasons for interruption of rooming-in**	
Newborn’s clinical reasons	43.08
Maternal rest	49.74
Both	7.18
**Quality of the information received**	
Clear and comprehensive	91.74
Need more information	7.02
**Bowel Movements and Diuresis**	
**Quality of the information received**	
Clear and comprehensive	92.9
Need more information	7.1
**Breastfeeding Observation**	
**Maternal general conditions**	
Relaxed and at ease	89.78
Tense and uncomfortable	9.49
Mixed feelings	0.73
**Neonatal general conditions**	
Wellbeing	90.53
Uneasiness	1.40
A combination of the two	8.07
**Newborn’s position**	
Adequate	79.42
Not adequate	2.88
Not sure	17.70
**Latch-on**	
Adequate	75.45
Not adequate	7.59
Not sure	16.96
**Suction**	
Adequate	58.55
Not adequate	3.85
Not sure	37.61
**Breast Examination**	
**Breast conditions**	
No change observed	34.44
Normal changes observed	49.79
Abnormal changes observed	15.77
**Nipple pain**	
Yes	60.17
No	39.83
**Nipple redness**	
No	60.58
Faded	25.73
Marked	13.69

**Table 4:** Breastfeeding outcomes: comparison between Phase 1 and Phase 2.

Variable	Total Population (*n* = 460)	Phase 1 (*n* = 191)	Phase 2 (*n* = 269)	*p*
Exclusive breastfeeding at discharge, *n* (%)	349 (75.9%)	154 (80.6%)	195 (72.5%)	**0.04**
Exclusive breastfeeding at 48 h post-discharge, *n* (%)	347 (75.4%)	149 (78%)	198 (73.6%)	0.28

*p* values < 0.05 are highlighted in bold.

**Table 5:** NPST median scores: comparison between Phase 1 and Phase 2.

NPST	Total Population (*n* = 460)	Phase 1 (*n* = 191)	Phase 2 (*n* = 269)	*p*
Total score	4.05 (3.52–4.48)	4.05 (3.57–4.48)	4.05 (3.48–4.48)	0.78
Informational support	4 (3.33–4.44)	4 (3.44–4.44)	4 (3.33–4.44)	0.86
Emotional support	3.67 (3–4.33)	3.67 (3–4.33)	3.67 (3–4.33)	0.49
Appraisal/parental esteem support	4 (3.25–4.5)	4 (3.25–4.75)	4 (3.25–4.5)	0.75
Caregiving support	4.4 (3.8–4.8)	4.4 (3.8–4.8)	4.4 (3.8–4.8)	0.49

## Data Availability

The data presented in this study are available upon request to the corresponding author. The data are not publicly available, due to privacy restrictions.

## Abbreviations

BFHI	Baby-Friendly Hospital Initiative
IBCLC	International Board-Certified Lactation Consultant
NICU	Neonatal Intensive Care Unit
NPST	Nurse–Parent Support Tool
UNICEF	United Nations Children Fund
WHO	World Health Organization
